# Bladder Wall Segmentation and Characterization on MR Images: Computer-Aided Spina Bifida Diagnosis

**DOI:** 10.3390/jimaging8060151

**Published:** 2022-05-25

**Authors:** Rania Trigui, Mouloud Adel, Mathieu Di Bisceglie, Julien Wojak, Jessica Pinol, Alice Faure, Kathia Chaumoitre

**Affiliations:** 1 Institut Fresnel, Centrale Marseille, CNRS, Aix Marseille University, 13013 Marseille, France; rania.trigui87@gmail.com (R.T.); julien.wojak@fresnel.fr (J.W.); 2 Medical Imaging Service, North Hospital, Aix-Marseille University, 13015 Marseille, France; mathieu.dibi@hotmail.fr (M.D.B.); Kathia.chaumoitre@ap-hm.fr (K.C.); 3 Paediatric Surgery Department, APHM, La Timone Children Hospital, 13005 Marseille, France; Jessica.PINOL@ap-hm.fr (J.P.); Alice.FAURE@ap-hm.fr (A.F.)

**Keywords:** magnetic resonance imaging, bladder wall segmentation, texture analysis, sequential floating selection, optimization, classification

## Abstract

(1) Background: Segmentation of the bladder inner’s wall and outer boundaries on Magnetic Resonance Images (MRI) is a crucial step for the diagnosis and the characterization of the bladder state and function. This paper proposes an optimized system for the segmentation and the classification of the bladder wall. (2) Methods: For each image of our data set, the region of interest corresponding to the bladder wall was extracted using LevelSet contour-based segmentation. Several features were computed from the extracted wall on T2 MRI images. After an automatic selection of the sub-vector containing most discriminant features, two supervised learning algorithms were tested using a bio-inspired optimization algorithm. (3) Results: The proposed system based on the improved LevelSet algorithm proved its efficiency in bladder wall segmentation. Experiments also showed that Support Vector Machine (SVM) classifier, optimized by Gray Wolf Optimizer (GWO) and using Radial Basis Function (RBF) kernel outperforms the Random Forest classification algorithm with a set of selected features. (4) Conclusions: A computer-aided optimized system based on segmentation and characterization, of bladder wall on MRI images for classification purposes is proposed. It can significantly be helpful for radiologists as a part of spina bifida study.

## 1. Introduction

Spina bifida, a congenital malformation visible from birth and characterized by poor development of the nervous system and the spine, could have several consequences on various organs and can lead to a multiple handicap. In the United States, about 1427 babies are born every year suffering from spina bifida [1], whereas in India, spina bifida rates are significantly higher, at 1.9 per 1000 births [2]. According to “France Assos Santé”, which brings together 84 national associations campaigning for the rights of patients and users, the number of patients with spina bifida in France is estimated to be 25,000 [3]. Spina bifida generates complex and variable disabilities from one individual to another. Depending on its severity, symptoms and patient needs, its management falls under multiple medical and surgical specialties.

Spina bifida is a neuro-spinal pathology whose clinical–pathological consequence is characterized by damage to the lumbosacral nerves. This has multiple neurological consequences, including bladder involvement with regard to the innervation of the main muscle of the bladder, but also on the bladder sphincters. This dyssynergy between these different muscle groups is the cause of bladder malformations, with thickening and irregularity of the bladder walls visible on the bladder imaging. This parietal damage is in fact the result of an antagonistic effect between a pathological detrusor wanting to evacuate urine from the bladder, and hyperactive bladder sphincters preventing the expulsion of urine.

Hence, the interest of studying the bladder wall characterization in order to identify useful information supporting the medical team decision. Indeed, a spina bifida case considered severe by specialists requires surgery intervention, unlike non-severe cases.

In order to characterize the bladder wall and to differentiate different spina bifida cases, a segmentation step is firstly crucial after the pre-processing step.

For this purpose, several segmentation strategies have been published in the literature [4,5,6]. As is also known, segmenting the inner bladder border is much easier than the outer one [7]. This is why, in many previous publications, only the inner border is segmented using an algorithm, whereas the outer border is segmented manually. Nonetheless, an automatic or semi-automatic segmentation of the bladder wall is already proposed in some previous publications. Contour-based segmentation methods have shown their robustness through several previous studies, especially Level-set segmentation, which is widely applied in bladder wall segmentation. Actually, in [8,9], and to segment inner and outer bladder boundaries, the authors proposed an adaptative shape prior constrained directional level set model with the exploitation of the prior knowledge of region information and bladder wall minimum thickness. A minimum thickness constraint of 1 mm is adopted to avoid an overlap between the outer and the inner zero level set. An adaptative $T_{1}$-weighted images level set segmentation strategy is also proposed by Duan et al. in [7]. Authors propose a coupled level set framework (two collaborative level set functions with an adaptative clustering algorithm) able to segment the inner and the outer bladder wall borders simultaneously for thickness measurement. The proposed framework was also compared to the Chan-Vese LS model [5], a segmentation algorithm based on level sets and designed to segment objects without clearly defined boundaries. A unified expectation-maximization (EM) approach with coupled level-set (CLS) constraints and a novel adaptative Markov Random Field model was also proposed by Hao Han et al. in [10] in order to segment the bladder wall in magnetic resonance imaging-based virtual cystoscopy. CLS was developed in order to preserve the continuity of the wall surface and to reduce the influence of artefacts adjacent to the bladder. Siqi Chen and Richard J. Radke [11] have also proposed a level-set segmentation approach based on both shape and intensity of prior information characterizing the image information using the intensity distributions inside the contour, outside it and over the entire region. In another more recent study [12], Wenjun Chi et al. segmented the bladder wall in MRI using coupled level set methods. Both of $T_{1}$-W and $T_{2}$-W modalities were used to detect inner and outer boundaries. Thus, there are many level-set based segmentation studies that have shown promising and robust results. Level set was also combined with deep-learning neural network in some recent studies in order to segment the urinary bladder such as in [13], in which Kenny H. Cha et al. developed a computerized system for bladder segmentation. This system starts with a deep learning convolutional neural network (DL-CNN) helping in the differentiation between the inside and the outside of the bladder and providing a seamless mask useful for guiding level set algorithm used here only to refine the segmentation. Deep learning completed by level sets was also the segmentation strategy of Gordon et al. in [6,14]. In fact, aiming to detect the inner and outer bladder wall in CT urography, authors firstly construct a DL-CNN likelihood map used after that as an energy term in the energy equation of a cascade level set methods. Many recent studies also proposed segmentation approaches uniquely based on deep learning [4,15,16,17] providing important segmentation results, but deep learning approaches often require a large database to afford the most reliable results.

After a successful segmentation step eventually compared to a ground truth provided by specialists, the next step is generally bladder characterization, for that many feature categories could be computed from the region of interest already extracted.

In order to discriminate bladder tumors from normal wall tissues, Xu et al. in [18] used three-dimensional texture features computed from MRI data. A total of 58 features were extracted from each Volume of Interest (VOI) such as gradient, curvature maps, 3D Haralick features and Tamura features. Effectively, four groups of texture features are used in their study, features computed from gray level co-occurrence matrix (GLCM), gray level-gradient co-occurrence matrix (GLGCM), gray-level-curvature co-occurrence matrix (GLCCM), and Tamura features. Texture features were also used in [19], by Shi et al. in order to characterize bladder carcinoma and bladder wall based on the exploration of textural characteristics on MRI data. Five categories of texture features were extracted, namely features based on the probability distribution of image intensity, Tamara features, features based on auto-covariance coefficients, features derived from GLCM and GLGCM matrices.

To the best of our knowledge, a complete segmentation, characterization and classification system for spina bifida staging using MRI data was developed in few research projects. As an example, we can cite the study of Khene et al. [20], in which contrast-enhanced Computed Tomography (CT) scans texture parameters, related to gray-level histogram and gray-level co-occurrence matrix, were used by the authors as urodynamic features for a spina bifida study in adult patients. Nevertheless, among the limitations of this study, bladder wall is segmented manually, and a few texture features were evaluated using “MaZda” tool [21].

**Figure 1:**
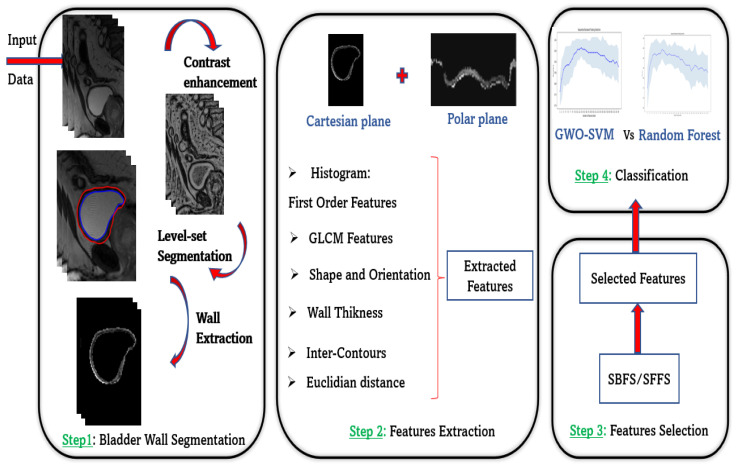
Overview workflow of our bladder wall characterization strategy.

This manuscript’s major objective is to propose a new semi-automated Computer Aided Diagnosis (CAD) System for accurate spina bifida staging classification on MRI images based on LevelSet bladder wall segmentation followed by feature extraction. Two automatic feature selection algorithms SBFS and SFFS are then used to determine the sub-vector containing the most significant features based on a sequential search strategy (more details in Section 2.3.2). These significantly different features are then analyzed using Support Vector Machine and Random Forest classifiers in order to determine their accuracy distinguishing severe spina bifida cases and non-severe ones as shown in Figure 1 below:

The remainder of this paper is organized as follows: first, details about the study data are provided. Next, the bladder wall segmentation methodology is described. Then, the proposed characterization strategy and employed features are fully explained. After that, experimental results are outlined and finally, discussion and conclusions are given.

## 2. Materials and Methods

### 2.1. Bladder MRI Data and Acquisition Techniques

This research project is part of a cooperation with the North Marseille Hospital, Timone children CHU and Conception Hospital CHU, Marseille, FRANCE. After approval by the Ethics Committee, 35 patients equivalent to 176 images were included in our study. Inclusion criteria were over 50 years of age patients requiring pelvic MRI for benign prostatic hypertrophy, consecutively included in the cohort. Exclusion criteria were a history of pelvic surgery (including bladder or prostate surgery) or vesical neoplasia.

All the analyses were performed on T2-weighted sagittal acquisitions using a 1.5-Tesla MRI scanner (Magnetom Amira, Siemens, Erlangen, Germany) with the following parameters: repetition time/echo time: 3630/90 ms; slice thickness: 3 mm; flip angle: 90°; matrix size: 448 × 336 mm^2^, field of view: 320 × 320 mm^2^. A T2-weighted sequence without fat saturation was used for two main reasons. On the one hand, it is a widely studied sequence used in clinical practice, on the other hand its characteristics allow a good resolution of contrast between urine, detrusor muscle and peri-vesical fat. The segmentation and annotation were done by radiologist with 6 years of experience. Each image was anonymized, collected, and stored for further processing in the digital imaging and communication in medicine (DICOM) format.

The use of the criterion of prostatic hypertrophy as an indication to perform MRI allows to classify patients in two classes: positive class and negative class regarding the thickening of their bladder walls. Positive class bladders were those in which obstructive pathological bladder wall was diagnosed on MRI acquisition. The remnographic criteria for a pathological bladder were present of at least one bladder diverticulum or irregular thickening of the bladder walls. A regular circumferential thickening could also be considered as pathological if it had a significant supra-physiological value (>6 mm). The presence of a focal thickening was not considered to be an obstructive pathological bladder, because of the insufficient spatial resolution of a 3-mm-thick MRI acquisition, and the possibility of the presence of a thickening that was tumoral and not related to obstructive thickening. Negative class bladders were those in which no focal or diffuse thickening of the bladder walls was demonstrated. The 176 acquisitions were divided into 97 images for negative class and 79 images for positive class.

Given that this study included patients with prostatic hypertrophy whose bladder wall thickness depends on the urine volume, a water-drinking protocol is followed during patient preparation. For adult patients, they are usually made to drink a 50 cL bottle one hour before the examination after urination. For these patients, bladder filling after drinking will depend on two main factors: more or less complete bladder emptying during their urination, and renal function and therefore the speed of “creation” of urine which is variable for each person. Concerning children, before each MRI, urological surgeons carried out a warm saline filling of their bladder to 80% of their theoretical bladder capacity in mL (corresponding to (age + 1) + 30). Urologists leave the bladder tube in place and perform a first sequence to check that they could clearly see the bladder, then T2 sequences in the 3 planes and 3D sequence.

### 2.2. Bladder Wall Segmentation

In T2-weighted MR images, the bladder wall has low-signal intensity and its appearance is different from the high-signal intensity urine and peri-vesical fat’s. If the images are not severely influenced by noise, the inner boundary can be identified correctly based on the large intensity gradient of active contour methods [22]. In this paper, a deformable model is proposed to segment the bladder wall. It can effectively handle the variation of intensity in MR images.

Therefore, in order to obtain a mask of the bladder wall that will be used for characterization and determination of spina bifida gravity, we have developed our algorithm which, based on the LevelSet, makes it possible to obtain the inner contour followed by the outer contour of the bladder, taking into account a certain neighborhood of each pixel.

- Overview of Level Set Method

LevelSet (LS) method, commonly used in the literature as contour based segmentation, was initially proposed in 1988, as a geometric deformable model, by Osher and Sethian [23], in order to implicitly describe evolving surfaces in 3D (or curves in 2D). Mathematically, let Ω ⊂ $R^{N}$ be the data domain, an N dimensional surface C is implicitly expressed by a scalar Lipschitz continuous function:

$$\phi \left(x_{1},x_{2},\ldots ,x_{n}\right):\;{\Omega \to R}^{N}$$

The contour evolution can be described by:

(1) $$\frac{d\phi }{dt}+F\left|\nabla \phi \;\right|=0$$

Here, |·| is the Euclidean norm and the nabla symbol ∇ represents the gradient.

*F* = $F^{image}$ + $F^{geometry}$; $F^{image}$ is the image force and $F^{geometry}$ is the geometry force [7,24].

The length of C with LS ϕ is represented as,

(2) $$\mathrm{Length}\;\{C\}=\int_{\Omega }^{}|\nabla H(\phi \left(x,y\right)|dxdy\;=\int_{\Omega }^{}\delta \left(\phi \left(x,y\right)\right)|\nabla (\phi \left(x,y\right)|dxdy\;$$

Such as (x,y) is the coordinates, δ(z) is the Dirac delta function and *H*(z) represents the Heaviside function [24] described as follows:

(3) $$H\left(z\right)=\{\begin{array}{c} 1,\;z\;\geq \;0\\ 0,\;z<0\;\left(3\right) \end{array}\;$$

- Proposed Segmentation Approach

**Figure 2:**
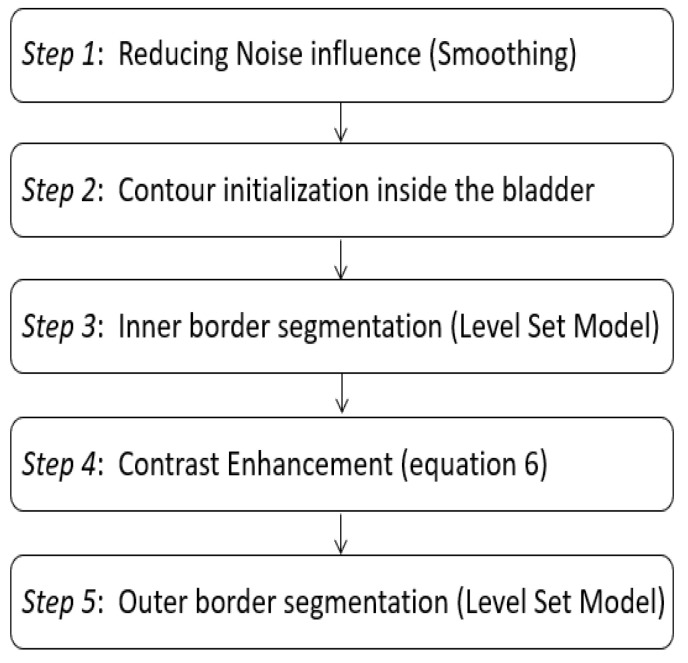
Overview of the full proposed segmentation procedure.

To start the treatment of the bladder wall, we need to reduce the noise influence. For that purpose, we smoothed every image in our data set by applying the basic morphological operations, dilation and erosion as shown in Figure 2 below. We specify that morphological operations were used only to facilitate segmentation. Moreover, we took care not to lose the information by extracting the attributes, used after that during the classification, from the non-denoised images.

After that, we initialize the Inner Level-Set Function (ILSF), used for the inner bladder wall border segmentation, inside the bladder lumen. This ILSF evolves until matching the inner border by LS model.

Then, and so as to facilitate and improve the outer boundaries detection, we enhanced the contrast of the image using the neighborhood pixel values (Equation (4)) and standardized the wall intensity knowing that the intensity of the bladder wall between the inner and the outer boundaries is usually homogeneous [8,9].

The initialization used for wall’s external contour segmentation, is the result of the internal contour segmentation.

Assuming that usually bladder wall thickness does not exceed a few pixels and that the outer border has generally a similar shape as the inner one, apart from some abnormalities, we have limited search area for the outer border taking into account the maximum wall thickness (1 mm) [8,9,25].

(4) $$I_{n}\left(x,y\right)=\;\frac{I_{i}\left(x,y\right)-M_{1}}{M_{2}-M_{1}}\cdot M_{x}$$

where $I_{i}\left(x,y\right)$ stands for the gray pixel intensity in the original image; $I_{n}\left(x,y\right)$ is the pixel intensity in the new contrast enhanced generated image; $M_{x}$ is the maximum gray level value of the original image; $M_{1}$ and $M_{2}$ are, respectively, the minima and the maxima of the initial image among a neighborhood pixel in a window of 9 × 9. When $M_{1}$ is equal to $M_{2}$, otherwise, in the case of a region of constant intensity, we keep the intensity $I_{i\;}\left(x,y\right)$.

### 2.3. Classification Methodology

#### 2.3.1. Global Feature Vector Construction

In order to identify characteristic image features that could reflect the difference between severe or not-severe spina bifida cases, masks of regions of interest (ROIs) were first applied to the T2 MRI dataset to extract only the wall. From this bladder wall, we extracted several attributes from cartesian and also from polar coordinates.

- Features based on the probability distribution of image intensity: five features

Using pixel gray level values of each segmented bladder wall, texture analysis also consists of calculating first order statistical parameters. Indeed, from the histogram and the cumulative histogram representing the segmented region, we computed different features, namely, the mean ($f_{0}$), the variance ($f_{1}$), the “kurtosis” ($f_{2}$), the “skewness” ($f_{3}$) and the area under cumulative histogram ($f_{4}$).

Skewness, effectively, measures the asymmetry of an histogram such as a zero value is an indication of a symmetrical distribution around the mean [20]. We suppose that, in thickened bladders that are poorly compliant, histograms are generally more skewed then the normal bladders characterized by less heterogeneous tissues [20,26].

Among the histogram-based features, we can also effectively calculate kurtosis, which describes histogram flatness. Thus, we speculate that an increased kurtosis is usually related with the bladder wall increased intensity variation, which can indicate microstructural changes [20,27].

- Textural features derived from the gray level cooccurrence matrix (GLCM): twenty features

Based on extracted ROIs, several texture features were computed using the GLCM matrix (Haralick features) in order to explore textural characteristics within different types of tissues. GLCM is effectively used to calculate the second order texture information. Therefore, we first need to create a Gray-Level Co-Occurrence Matrix in which each element represents the probability of two pixels with a given gray level intensity and separated by a given distance [19]. From this matrix several statistics can be derived after offsets specifications. Based on GLCM matrix, a total of the 20 features were computed in this study as described in Table A1 in the Appendix A.

- Features based on the wall Shape and Orientation: 9 features

After the delimitation of the internal and the external contours of the bladder wall, we computed nine morphological parameters of shape and orientation, based on the external mask, as described in Table A2 in the Appendix A.

- Wall Thickness in polar coordinates: 1 feature ($f_{34})$

**Figure 3:**
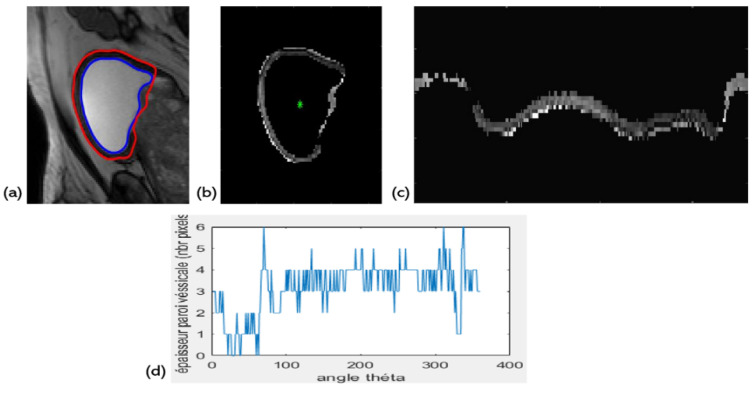
Exploration of the polar representation: (**a**) Level set bladder wall segmentation. (**b**) Bladder wall extraction in cartesian coordinates. (**c**) Switching to polar coordinates: θ angle on the *x*-axis, radius R on the *y*-axis. (**d**) The wall thickness as a function of the angle θ.

Several studies have shown that bladder wall thickness can be a meaningful measurement for medical bladder characterization. The normal bladder is effectively usually thin and smooth if it is moderately distended [22]. For that reason, we are interested in this study by evaluating the thickness of the wall as a function of the angle θ in polar coordinates as shown in Figure 3 below.

To obtain a representation of the MR image in polar coordinates, we started by determining the bladder’s center of mass, denoted by *C* =$\;{\left[C_{x},\;C_{y}\right]}^{T}$ ∈ ${\mathbb{R}}^{2}$. Then, the intensity of a pixel (*r*, θ) in the image in polar coordinates is:

(5) $$I_{p}\;(r,\;\theta )=I(x,y)$$

where the pixel position correspondence is given by

(6) $$X=C_{x}+r\;\cos\;\theta \;y=C_{y}+r\;\sin\;\theta$$

- Boundaries similarity: 5 features

**Figure 4:**
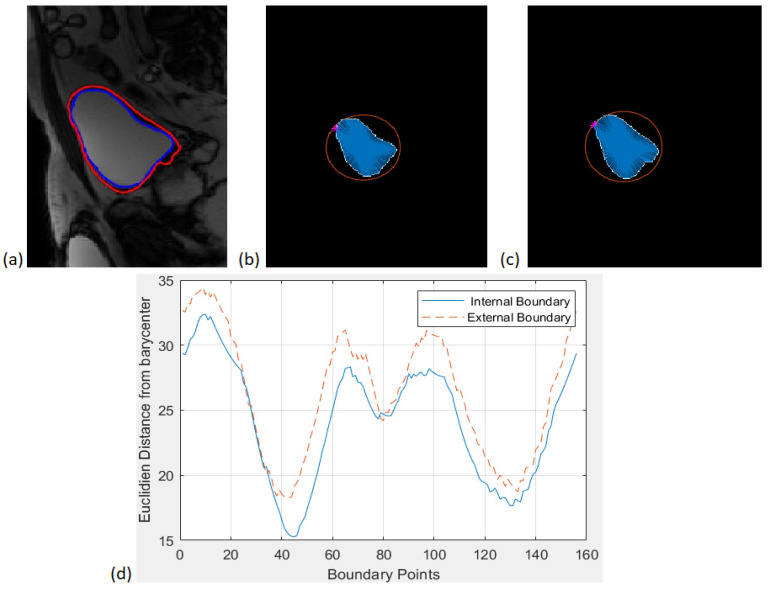
(**a**) An example of LevelSet bladder wall segmentation. (**b**) Matching the bladder’s barycenter with each point of the internal boundary. (**c**) Matching the bladder’s barycenter and each point of the external boundary. (**d**) Euclidean distance between the barycenter and both of the inner and outer contours.

Since spina bifida disease can be characterized by deformities on the bladder wall, we also thought of representing the shape of the two contours by measuring the Euclidean Distance between the center of mass of the bladder and each point of the contours in order to characterize the similarity between the inner and the outer bladder contour shapes as shown in Figure 4 below. An interpolation have be done in order to represent the two curves in the same coordinate system with the same number of points.

Once we have plotted the two curves corresponding to the inner and the outer boundaries of the bladder, we calculate five attributes as follows:

✓ Difference of areas under the two curves ($f_{35}$);
✓ Sum of Squared Difference between the two curves ($f_{36}$);
✓ Sum of Absolute Difference between the two curves ($f_{37}$);
✓ Mean distances: Average difference for the same abscissas ($f_{38}$);
✓ Max Distance: Maximum distance between the two curves in point-to-point comparison for the same abscissas ($f_{39}$).

A total of 40 attributes are computed and concatenated in an attribute vector X used as input for two supervised classifiers: support vector machine and Random Forest after a crucial step, which is automatic feature selection.

#### 2.3.2. Feature Selection

Each extracted attribute can be suitable for a well-defined application providing a strong power of discrimination between different classes. However, none can be absolutely effective combined with other attributes in all types of applications. Moreover, the presence of some attributes in the input vector fed to the classifier can even degrade classification efficiency.

Thus, so as to optimize the classification error and reduce the processing time, we have chosen to use a sequential search strategy by applying two automatic feature selection algorithms SBFS “Sequential Backward Floating Selection” and SFFS “Sequential Forward Floating Selection” [22].

SFFS start from the empty set $Y_{0}\;$= {ø} and sequentially ass a feature $X^{+}$ that gives the highest performances (accuracy) when it is combined with the already selected sub-set of features $Y_{k}$ until all 40 attributes are tested.

SBFS algorithm starts with the full set of extracted attributes (size = 40), and sequentially removes the feature $X^{-}$ related to the smallest decrease in the objective function’s value; in other words, we remove the feature that gives the lowest performance [22,28].

Regarding our application, we chose to test all the possible combinations with the evaluation of classification accuracy based on two supervised classification algorithms.

#### 2.3.3. Gray Wolf Optimization-Support Vector Machine (GWO-SVM)

- SVM Classifier

SVM is a supervised machine learning classifier successfully used in medical field. It was introduced in 1990 by Vladimir Vapnik [26]. The basic principle of this classifier is to search the hyperplane that separates two classes, maximizing the margin between them [29,30]. This margin represents the Euclidean distance between the optimal hyperplane and the nearest point of the training set. In case of non-linearly separable data, SVM transforms the data representation space into a one of larger dimension.

The SVM decision rule is [31]:

(7) $$f\left(x\right)=\mathrm{sign}\left(\overset{N}{\underset{i=1}{\sum }}\alpha_{i}y_{i}K\left(x_{i},x\right)+b\right)$$

where ($x_{1},x_{2},\ldots ,x_{N}$) vectors in space $x_{i}$ ∈ ${\mathbb{R}}^{d}$ with their labels ($y_{1},y_{2},\ldots ,y_{N}$) such as $y_{i}$∈ ${\left(+1,-1\right)}^{N}$. α = ($\alpha_{1},\ldots ,\alpha_{N}$) represent the *N* non-negative Lagrange multipliers, *b* is a bias, *x* represents the input vector and *K* is the Kernel function.

With SVM classifier we can use a kernel function among the existing different types: linear, polynomial, Gaussian Radial Basis Functions (RBF), etc. The most widely used is RBF because of its capacity to deal with highly nonlinear training data and because mathematically; it is less complex then the polynomial kernel for example [32]. The Gaussian RBF kernel can be arithmetically denoted as:

(8) $$K\left(x_{i},\;x_{j}\right)=\exp\;(\frac{||x_{i}-x_{j}|{|}^{2}}{2\;\gamma^{2}})$$

where γ is the thickness of the gaussian RBF kernel function [32].

- Grey Wolf Optimizer (GWO)

SVM has been shown to be effective for different applications. Nevertheless, good performances depends on the choice of the most adequate values of its parameters. Testing different possible pair of parameters is an exhaustive operation. This is why there is an increasing tendency to use automatic optimization algorithms.

According to literature, Gray Wolf Optimization (GWO) algorithm is the best one that makes balance between exploration or global research and exploitation or local research [33]. It has also simple structure, fewer parameters and strong and rapid convergence compared to other bio-inspired algorithms like particle swarm optimization (PSO) or genetic algorithm (GA) [34].

GWO is a new meta heuristic technique and swarm intelligence algorithm that was first proposed by Mirjalili et al. [33] and adapted to estimate SVM parameters for different applications such as the classification of color image [35] and gender classification [36]. Nevertheless, although it has been used for some medical applications, to the best of our knowledge, it has never been applied for bladder wall characterization.

This optimization algorithm, adopted to locate the best position or solution in a fixed search space, is inspired from the grey wolves’ hunting and searching behaviors and social hierarchy.

The best position or the first optimal solution is associated to alpha *α* gray wolf, located at the top food chain and responsible for decision making and leadership [37]. The second and third optimal solutions are, respectively, beta β and delta *δ*. The rest of the solutions are set to omega *ω*. During the search process, the wolfs position managed by the Formulas (9) and (10), updates constantly.

(9) $$D=|C\cdot X_{p}(t)-X(t)|$$

(10) $$X(t+1)=X_{p}(t)-A\cdot D$$

Such as *A* = 2 · a $\cdot \;r_{1}$ − a and C = 2 · $r_{2}$. Where *t* is the current iteration, $X_{p}\;\mathrm{is}\;\mathrm{the}$ prey position, *X* is the position of a gray wolf, $r_{1}$ and $r_{2}$ are random vectors and *a* linearly decreases from 2 to 0. Therefore, based on Formulas (9) and (10) we can calculate $D_{\alpha }$, $D_{\;\beta }$, $D_{\delta }$, $X_{1}$, $X_{2}$ and $X_{3}$ corresponding to alpha, beta and delta wolves, respectively. Then, the final position of wolves *ω* is defined by Formulas (11):

(11) $$X(t+1)=|\frac{\left(X_{1}+X_{2}+X_{3}\right)}{3}|$$

Thus, SVM parameters C (penalty factor) and γ (associated to the RBF kernel) can be optimized thanks to the GWO optimization algorithm after *n* iterations and updates.

**Figure 5:**
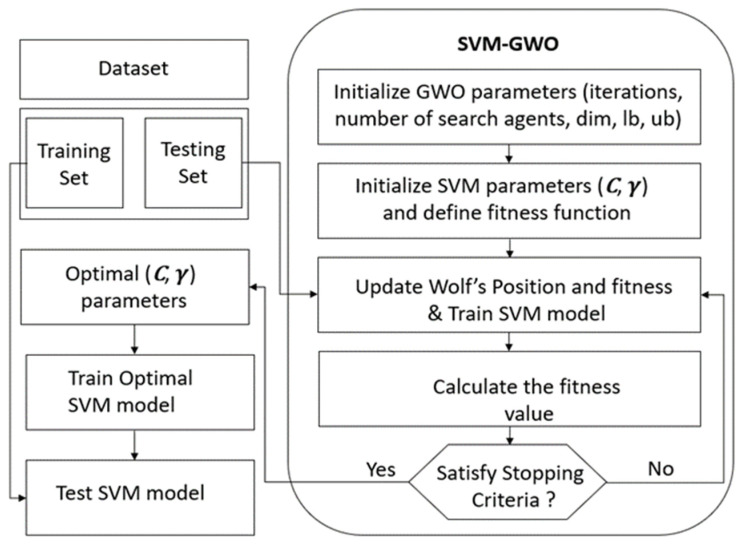
The flowchart of our proposed GWO-SVM algorithm.

For that we first start by selecting a part of dataset as the training set and use the remaining feature vector for test step in order to verify SVM recognition accuracy. After that, we initialize GWO parameters such as the number of wolf packs, the number of iterations and the research space dimensions. The goal is to maximize the accuracy. Global optimal SVM parameters found by GWO correspond to the outputs when reaching the maximum number of iterations as shown in Figure 5.

#### 2.3.4. Random Forest

Decision trees are among the most popular supervised learning-based classification that have been widely adopted to solve problems in the medical image processing field [4,13,15]. In 2001, Leo Breiman proposed an extension of decision trees with random forests [38,39], made up of a set of decision trees where each of these trees is interested in a random subset of samples. In this paper we have proposed to compare the classification performances of the random forests algorithm and those of the GWO-SVM approach.

## 3. Results

### 3.1. Segmentation Results

In order to characterize the bladder wall, we first started with its segmentation. For that purpose we used the semi-automatic contour based segmentation algorithm: LevelSet. Our segmentation strategy was already described with details in Section 2.2.

Thanks to the good contrast between the bladder lumen and the wall, the inner border is firstly and generally easily segmented based on the intensity contrast information. However, outer border segmentation, is more complicated because of the variability in the contrast on the surrounding tissues.

**Figure 6:**
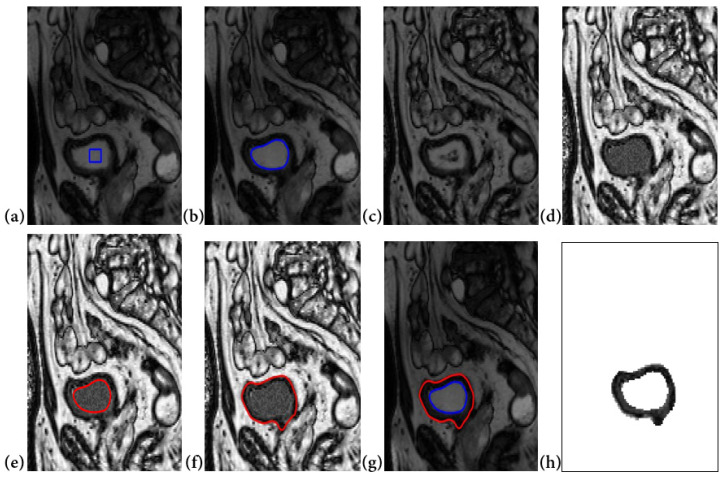
Segmentation procedure using our proposed algorithms: (**a**) Contour initialization inside the bladde, (**b**) the inner boundary segmented by the level set algorithm, (**c**) contrast enhanced generated image, (**d**) pixel intensity change inside the bladder according to the average intensity in the bladder wall, (**e**) inner segmentation result as initialization for the outer boundary research, (**f**) the outer boundary segmented, (**g**) inner and outer contours and (**h**) bladder wall extracted.

After the inner contour segmentation step, and in order to improve outer contour segmentation results, we proposed to generate a contrast enhanced image (Equation (4)) on which we extract the external contour. We also added some modifications on the LevelSet algorithm so as to limit the search area for the outer border since the wall thickness usually does not exceed a certain threshold value estimated as few pixels, as explained in Section 2.2 [8,9,25]. After contrast enhancement, we apply a pixel intensity standardization (Figure 6d), which consists on filling the region delimited by the internal contour of the bladder with gray level intensities between the maximum intensity and the minimum intensity of the bladder wall. Although Level Sets segmentation is not fully automated because it requires an initialization inside the bladder, which evolves until reaching the internal bladder wall, we have tried several sizes of initializations, which always gave the same results.

Segmentation results are illustrated in Figure 6 below.

**Figure 7:**
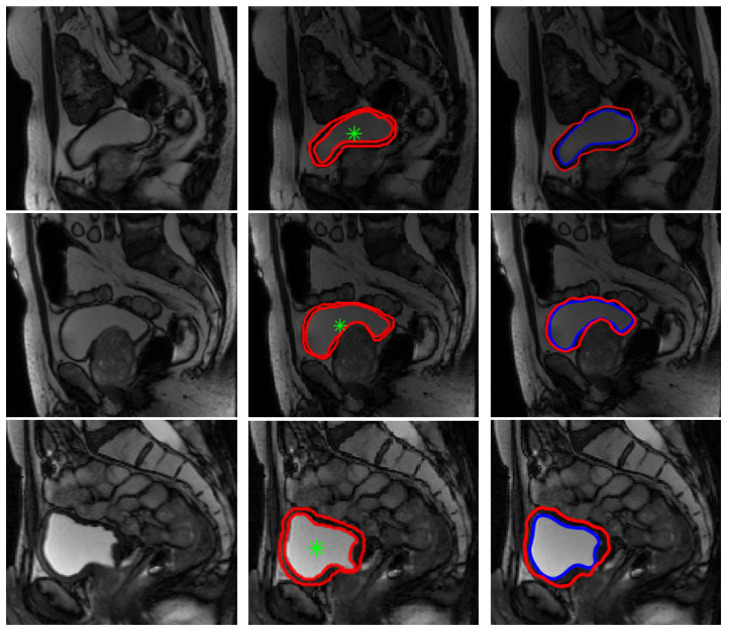

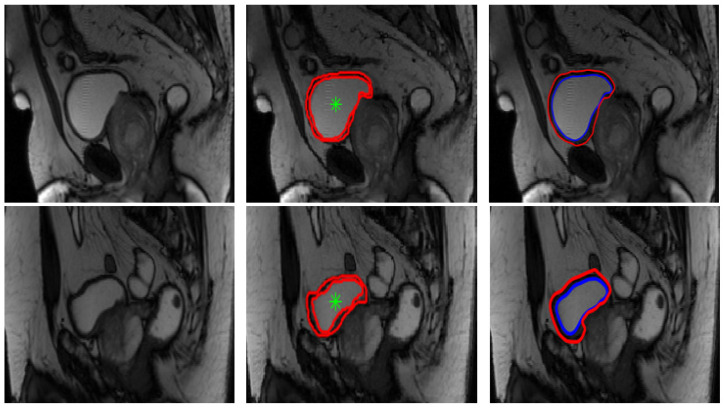
Examples of bladder wall segmentation results. **Left column**: original T2-weighted imaging. **Middle column**: bladder wall expert manual segmentation. **Right column**: Proposed Level Set approach segmentation results.

Segmentation results on our database were considered satisfactory by the medical team (some examples are presented in Figure 7 below). Manual segmentation considered as a ground truth was done by the medical team using a matlab algorithm that we have developed to be used by radiologists in our laboratory. As medical image segmentation is a crucial preprocessing step, comparing the quality of the semi-automatic segmentation with ground truth is an essential part. Therefore, in order to get a statistical validation, we have computed some metrics usually used for evaluating medical image segmentation, namely, Dice coefficient (DICE), mutual information and mean overall error rate, as shown in Table 1 below. The mutual information between two random variables [40] is defined as the measure of dependency between them. The Dice coefficient [41], called the overlap index and described in Equation (12), is the most used to validate medical segmentation:

(12) $$\mathrm{DICE}=\frac{2\left|S_{g}\cap S_{t}\right|}{\left|S_{g}\right|+\left|S_{t}\right|}$$

Such as $S_{g}$ represents the ground truth segmentation and $S_{t}$ is the segmentation to be evaluated.

Segmentation comparison results presented in Table 1, confirm the medical team validation and show the efficiency of our contour-based segmentation strategy. These good segmentation results allow as to have a reliable characterization thereafter.

It is important to notice that in this study, we used a local data base provided by the hospital, which explains the difficulty of comparing our results with other research carried out on other databases and under other conditions.

After the validation of our bladder wall segmentation results, we started the characterization by calculating 40 attributes described in Table A1 and Table A2 in the Appendix A (from $f_{0}\;\mathrm{to}\;f_{39}$). In order to test the feasibility of using statistically significant features for the differentiation of severe and not-severe spina bifida cases, classification study was further performed. The assessment of bladder involvement or not (severe or not severe data) was made by considering several elements, in particular the thickness of the bladder wall, the irregularity or otherwise of the wall, the presence of trabeculations, etc. These elements were interpreted by two radiologists specialists with 7 and 23 years of experience.

We prepared our features data to be fed into the classifier by normalizing values between −1 and 1 because features have different ranges. Two feature selection algorithms (SBFS and SFFS) were also used in order to select the best feature subset giving the best classification performances with SVM, GWO-SVM and Random Forest classifiers.

Given its high performances and its generalization capability, a support vector machine classifier was first used in this study. Moreover, for the kernel function, we compared RBF, linear and polynomial kernels. In fact, even if generally the gaussian RBF kernel performs better than other kernel types, in this study we have chosen to confirm it experimentally.

Overall, 80% of data was used in the training step and 20% for testing, and 5-fold- cross-validation procedure was followed. All features were effectively randomly divided into five subsets, so that the SVM classifier is trained by nine subsets and tested by the remaining subset. This procedure is repeated so that each of the five subsets is used once for testing. This procedure is so important and is able to ameliorate the classification performances.

In order to optimize SVM parameters using RBF kernel, we propose in this paper to apply the bio-inspired optimization method GWO. As output, our GWO function provides the best values of C and γ giving the best classification accuracy of the support vector machine.

**Figure 8:**
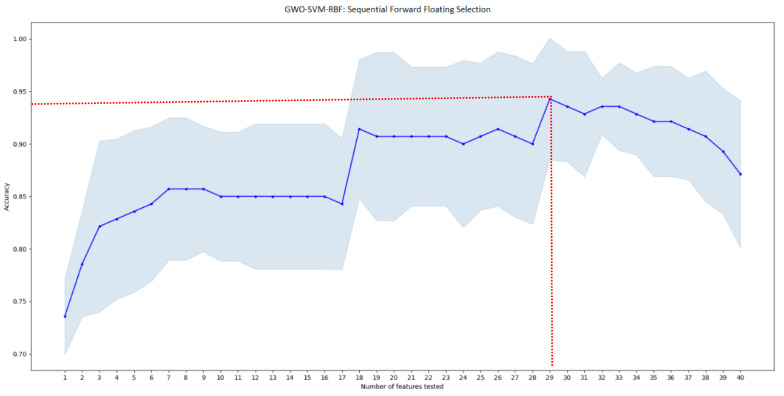
GWO-SVM classification accuracy score according to the sub-vector of tested features using SFFS algorithm. Red lines indicate the best performance achieved.

After an automatic feature selection of the most significative features using two different algorithms (SBFS and SFFS) and testing three types of kernels, SFFS selection algorithm with GWO-SVM classifier and RBF Kernel and a combination of 29 attributes gave the best accuracy score of 94.4% as shown in Table 2 and Figure 8.

### 3.2. GWO-Support Vector Machine Results

As explained previously, we applied GWO algorithm to optimize the SVM-RBF optimization. Results demonstrate that, to achieve the best performances (accuracy score of 94.4%, sensitivity of 94.74%, specificity of 94.12% and precision of 94.74%), GWO fixed the regularization parameter C = 9.6517 and gamma γ = 0.015.

The best sub-set of features giving the highest accuracy rate, englobe 14 GLCM texture attributes (contrast, prominence, dissimilarity, energy, entropy, homogeneity, maximum probability, variance, difference variance, difference Entropy, Information measure I of correlation, Information measure II of correlation, Inverse Difference Normalized, Inverse difference moment), mean, variance, kurtosis and area under cumulative histogram, six attributes representing shape and orientation (Area, circularity, eccentricity, solidity, roundness, axis ratio) and all of the five attributes describing the boundaries similarity.

### 3.3. Random Forest Results

**Figure 9:**
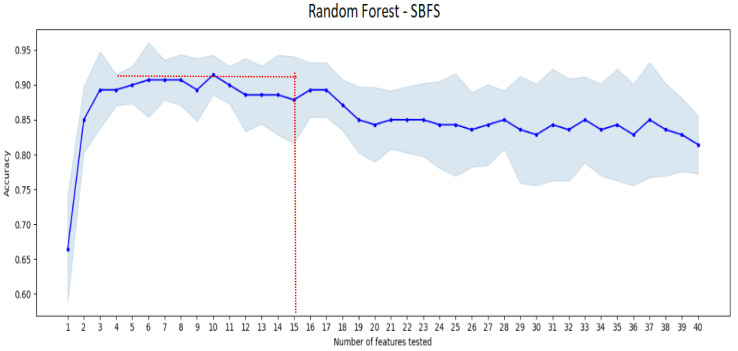
Random forest classification accuracy score according to the sub-vector of tested features using SBFS algorithm. Red lines indicate the best performance achieved.

After an automatic feature selection of the most significative features using two different algorithms, SBFS selection algorithm with Random Forest classifier and a combination of 10 attributes gave the best accuracy score for this classifier of 91.67% as shown in Figure 9 below.

The best sub-set of features giving the highest accuracy rate for the Random Forest classifier, a sensitivity of 94.44%, a specificity of 88.8% and a precision of 89.4%, englobe 4 GLCM texture attributes (contrast, correlation, difference variance, Inverse Difference Normalized), the variance of the associated histogram, four attributes representing shape and orientation (Area, orientation, perimeter, axis ratio) and the difference of areas under the two curves representing the inner and the outer boundaries of the bladder as shown in Table 3.

## 4. Discussion

The goal of the proposed approach is to provide an accurate segmentation and optimized characterization of the bladder wall in magnetic resonance imaging, which represents a computer-aided spina bifida diagnosis able to facilitate the radiologist’s decision.

First, results prove that the proposed segmentation method based on Level Set algorithm is able to give a good and efficient segmentation of the inner and the outer boundaries compared to the ground truth. Our segmentation protocol can be helpful for radiologists avoiding the fastidious traditional manual segmentation, especially for specific cases where bladder walls are so thin.

Several features computed from a segmented bladder wall in T2-W MRI adults dataset in cartesian and polar coordinates have been evaluated. The best classification performance was obtained with a combination of 29 attributes including texture, shape, orientation and boundaries similarities.

A comparison between three SVM kernel types proved that RBF kernel gives the best accuracy score. Results have also shown that SVM outperforms Random Forest classifier when using RBF kernel. It has also been proved that the optimization of SVM parameters using the bio-inspired algorithm GWO was useful increasing SVM classification accuracy from 91.67% to 94.4% when using RBF kernel and SFFS feature selection algorithm.

We chose, in this project, a contour-based segmentation instead of a deep learning model because, at the beginning of this project, we had a small data base provided by our medical staff. Moreover, conventional methods based on the extraction and selection of attributes and then classification are also chosen in this project because our objective is to understand the correlation between the calculated attributes and the spina bifida disease severity, which cannot be provided by deep learning models considered as black boxes.

Thus, using a classification protocol including two classes, we showed experimentally that it is possible to give an efficient discrimination tool sensitive to sufficiently reveal the differences between severe, non-severe spina bifida cases.

## 5. Conclusions

In this paper, we propose a new bladder wall segmentation strategy, which is efficient for diagnosis of spina bifida severity. The proposed approach, based on the Level Set algorithm with image contrast enhancing and limiting the search area, can effectively handle the inner and the outer boundaries segmentation and the effective characterization of the bladder wall. A GWO-SVM compared with Random Forest approach is also performed. The proposed method was applied to an MRI data base as a part of spina bifida study. Experimental results proved the ability of our characterization method to distinguish between severe and non-severe spina-bifida. Our evaluations also show that MRI texture analysis related to the gray-level histogram and GLCM matrix of bladder wall associated with other features describing the shape, orientation, wall thickness and boundaries similarities could be an interesting noninvasive tool in the identification of high risk spina bifida patients.

In our further work, we would like to test our method on a larger dataset so as to make using the deep learning more significative. We also would like to further investigate in the 3D bladder MRI segmentation with shape prior constraint, and to take into account new features as 3D textural features.

## Figures and Tables

**Table 1:** Comparison results between ground truth and level set segmentation.

Metric	Mean Dice	Mean Mutual Information	Mean Overall Error Rate
Value	0.826	0.801	0.262

**Table 2:** Support vector machine classification results using different kernels (the definition of the features can be found in Table A1 and Table A2 in the Appendix A).

Classification Method	Kernel Type	Feature Selection Algorithm	Accu.	Sensi.	Speci.	Preci.	Best Features Sub-Set
SVM	RBF	SFFS	0.9167	0.8947	0.9412	0.9444	$20\;\mathrm{attributes}\;\{f_{6},\;f_{7},\;f_{11},\;f_{12},\;f_{13},\;f_{14},\;f_{21},\;f_{22},f_{0},\;f_{1},\;f_{25},\;f_{26},f_{28},\;f_{30},\;\;f_{32},\;f_{33},\;f_{34},\;f_{35},\;f_{37},\;f_{38}\}$
		SBFS	0.9167	0.9	0.9375	0.9474	$9\;\mathrm{attributes}\;\{f_{7},\;f_{14},\;f_{22},\;f_{28},\;f_{29},\;f_{32},\;f_{33},\;f_{35},\;f_{38}\}$
GWO-SVM	RBF	SFFS	0.944	0.9474	0.9412	0.9474	$29\;\mathrm{attributes}\;\{f_{6},\;f_{8},\;f_{10},\;f_{11},\;f_{12},\;f_{13},\;f_{14},\;f_{15},f_{19},\;f_{20},\;f_{21},\;f_{22},f_{23},\;f_{24},\;\;f_{0},\;f_{1},\;f_{2},\;f_{25},\;f_{26},\;f_{27},\;f_{30},\;f_{31},\;\;f_{33},\;f_{4},\;f_{35},\;\;f_{36},\;f_{37},\;f_{38},\;f_{39}\}$
		SBFS	0.9394	0.9412	0.9375	0.9412	$21\;\mathrm{attributes}\;\{f_{6},\;f_{9},\;f_{10},\;f_{11},\;f_{14},\;f_{17},\;f_{19},\;f_{20},f_{21},\;f_{22},\;f_{23},\;f_{0},f_{3},\;f_{25},\;\;f_{26},\;f_{27},\;f_{28},\;f_{31},\;f_{32},\;f_{34},\;f_{36}\}$
SVM	Linear	SFFS	0.9167	0.9375	0.9	0.884	$25\;\mathrm{attributes}\;\;\{f_{6},\;f_{7},\;f_{9},\;f_{10},\;f_{11},\;f_{12},\;f_{13},\;f_{15},f_{16},\;f_{17},\;f_{18},\;f_{19},f_{20},\;f_{21},\;\;f_{23},\;f_{2},\;f_{25},\;f_{28},\;f_{31},\;f_{32},\;f_{33},\;f_{4},\;f_{36},\;f_{37},\;f_{39}\}$
		SBFS	0.8889	0.944	0.833	0.85	$19\;\mathrm{attributes}\;\;\{f_{6},\;f_{7},\;f_{9},\;f_{11},\;f_{13},f_{17},\;f_{18},\;f_{19},\;f_{20},f_{21},\;f_{22},\;f_{0},\;f_{1},f_{27},\;f_{28},\;\;f_{29},\;f_{37},\;f_{38},\;f_{39}\}$
	Pomynomial	SFFS	0.833	0.904	0.733	0.826	$9\;\mathrm{attributes}\;\;\{f_{10},\;f_{13},\;f_{22},\;f_{23},f_{1},\;f_{25},\;f_{30},\;f_{31},\;f_{39}\}$
		SBFS	0.805	0.8333	0.77	0.7895	$4\;\mathrm{attributes}\;\{f_{13},\;f_{16},\;f_{29},\;f_{33}\}$

**Table 3:** Random forest classification results (the definition of the features can be found in Table A1 and Table A2 in the Appendix A).

Classification Method	Feature Selection Algorithm	Accu.	Sensi.	Speci.	Preci.	Best Features Sub-Set
Random Forest	SFFS	0.8889	0.8947	0.8824	0.894	$8\;\mathrm{attributes}\;\{f_{2},\;f_{9},\;f_{16},\;f_{25},\;f_{27},\;f_{28},\;f_{34},\;f_{38}\}$
	SBFS	0.9167	0.9444	0.888	0.894	$10\;\mathrm{attributes}\;\{f_{1},\;f_{2},\;f_{14},\;f_{18},\;f_{21},\;f_{24},\;f_{27},\;f_{28},\;f_{32},\;f_{35}\}$

## Data Availability

We use in this project a local dataset which is not publicly archived.

## Appendix A

**Table A1:** Definition of gray-level co-occurrence matrix (GLCM) descriptors.

Feature Group	Feature Id	Feature Description
GLCM	$f_{5}$	Auto-Correlation
	$f_{6}$	Contrast
	$f_{7}$	Correlation
	$f_{8}$	Prominence
	$f_{9}$	Shade
	$f_{10}$	Dissimilarity
	$f_{11}$	Energy
	$f_{12}$	Entropy
	$f_{13}$	Homogeneity
	$f_{14}$	Maximum Probability
	$f_{15}$	Variance
	$f_{16}$	Sum Average
	$f_{17}$	Sum Variance
	$f_{18}$	Sum Entropy
	$f_{19}$	Difference variance
	$f_{20}$	Difference Entropy
	$f_{21}$	Information measure I of correlation
	$f_{22}$	Information measure II of correlation
	$f_{23}$	Inverse Difference Normalized
	$f_{24}$	Inverse difference moment

**Table A2:** Details of the computed morphological parameters.

Attribute	Explication
$\mathrm{Area}\;(f_{25}$)	Number of pixels in the studied region.
$\mathrm{Circularity}\;(f_{26}$)	This parameter compares the ROI shape and a cercle. It is computed as follows: $C=\frac{4\Pi \;x\;Area}{Perimeter^{2}}$
$\mathrm{Eccentricity}\;(f_{27})$	Eccentricity of the ellipse with the same second moments as the region.
$\mathrm{Orientation}\;(f_{28})$	Represented by the angle between the x-axis and the maximum diameter of the best ellipse having the same second-moments as the region.
$\mathrm{Perimeter}\;(f_{29})$	Distance around the region’s boundary.
$\mathrm{Solidity}\;(f_{30})$	$\mathrm{Solidity}=\frac{Mask\;Area}{Area\;of\;the\;convex\;envelope}$
$\mathrm{Roundness}\;(f_{31})$	This factor is used to describe the shape and can be represented by this formula: $\frac{4\;\times \;Area}{\Pi \;\times \;maximum\;diameter^{2}}$ -Roundness is close to 1 with circular shape -Roundness is close to $\frac{r_{1}}{r_{2}}$ in the case of elliptical shape -Otherwise: irregular shape Such as $r_{1}$$\;\mathrm{and}\;r_{2}$ represent respectively half of the minor axis and the major axis
$\mathrm{ENC}\;(f_{32})$	Elliptical Normalized Circumference: We compare the bladder mask’s shape with ellipse closest to its shape. $\mathrm{ENC}=\frac{Best\;ellipse\;perimeter}{ROI\;perimeter}$
Ratio between the major and the minor axis lengths $\left(f_{33}\right)$	This Ratio between the maximum width and the maximum length characterizes the external bladder shape.
